# BRCA1 modulates the autophosphorylation status of DNA-PKcs in S phase of the cell cycle

**DOI:** 10.1093/nar/gku824

**Published:** 2014-09-15

**Authors:** Anthony J. Davis, Linfeng Chi, Sairei So, Kyung-Jong Lee, Eiichiro Mori, Kazi Fattah, Jun Yang, David J. Chen

**Affiliations:** 1 Division of Molecular Radiation Biology, Department of Radiation Oncology, University of Texas Southwestern Medical Center, 2201 Inwood Rd, Dallas, TX 75390, USA; 2 The First Affiliated Hospital, State Key Laboratory for Diagnosis and Treatment of Infectious Diseases, Zhejiang University, Hangzhou, Zhejiang, China; 3 Department of Toxicology, Hangzhou Normal University School of Public Health, 16 Xue Lin Street, Hangzhou, Zhejiang, China

## Abstract

Non-homologous end-joining (NHEJ) and homologous recombination (HR) are the two prominent pathways responsible for the repair of DNA double-strand breaks (DSBs). NHEJ is not restricted to a cell-cycle stage, whereas HR is active primarily in the S/G2 phases suggesting there are cell cycle-specific mechanisms that play a role in the choice between NHEJ and HR. Here we show NHEJ is attenuated in S phase via modulation of the autophosphorylation status of the NHEJ factor DNA-PKcs at serine 2056 by the pro-HR factor BRCA1. BRCA1 interacts with DNA-PKcs in a cell cycle-regulated manner and this interaction is mediated by the tandem BRCT domain of BRCA1, but surprisingly in a phospho-independent manner. BRCA1 attenuates DNA-PKcs autophosphorylation via directly blocking the ability of DNA-PKcs to autophosphorylate. Subsequently, blocking autophosphorylation of DNA-PKcs at the serine 2056 phosphorylation cluster promotes HR-required DNA end processing and loading of HR factors to DSBs and is a possible mechanism by which BRCA1 promotes HR.

## INTRODUCTION

DNA double-strand breaks (DSBs) are the most deleterious type of DNA lesion because if unrepaired or misrepaired they can promote chromosomal aberrations resulting in genomic instability, which is a driving force for tumorigenesis (1). The cellular response to DSBs is extensive and includes recognition of the DNA lesion, signal transduction responses including modulation of the cell cycle and finally repair of the DSB (2,3). There are two prominent DSB repair pathways in eukaryotic cells termed homologous recombination (HR) and non-homologous end-joining (NHEJ). HR mediates DSB repair via use of a homologous DNA sequence as a template to guide proper restoration of the broken DNA molecule. The HR pathway starts following recognition of the DSB by the Mre11/Rad50/Nbs1 (MRN) complex and initiation of 5′-3′ resection of the DSB by MRN, CtIP and exonuclease 1 (4,5). DNA end resection produces 3′ ssDNA overhangs that are bound and stabilized by Replication Protein A (RPA). Subsequently, RPA is replaced on the ssDNA by Rad51 and strand invasion and exchange into a homologous DNA template occurs. Following DNA synthesis, ligation and branch migration, the recombination intermediates are resolved and the break is repaired. NHEJ is characterized by its ability to directly ligate the two ends of the broken DNA molecule (6,7). NHEJ is initiated by the association of the Ku70/80 (Ku) heterodimer to DNA ends where it then functions primarily as a scaffold to recruit the NHEJ machinery to the DSB. One of the primary factors Ku recruits to the DSB is the DNA-dependent protein kinase catalytic subunit (DNA-PKcs). Recruitment of DNA-PKcs to the DSB mediates the formation of the DNA-PK complex (Ku70/80 with DNA-PKcs) and results in activation of its catalytic activity, which is required for NHEJ. Subsequently, DNA-PKcs is phosphorylated and autophosphorylated at a number of sites in response to DNA damage with the best characterized being the serine 2056 and threonine 2609 phosphorylation clusters (7). Phosphorylation of these two clusters is important for NHEJ as ablation of either phosphorylation cluster causes increased radiosensitivity and less efficient DSB repair (8–12). Finally, if required, the two DNA termini are processed and finally ligated by the XRCC4-DNA Ligase IV complex.

Since there are multiple DSB repair processes, a cell must properly choose which pathway to employ for each specific DSB. A number of mechanisms have been proposed to play a role in DSB repair pathway choice. It has long been speculated that choice between NHEJ and HR may simply reside via competition between the NHEJ and HR DNA damage sensor proteins for binding to the DSB (13). The choice of HR over NHEJ is influenced by the cell-cycle stage as HR is thought to primarily be active during S and G2 phases of the cell cycle when a homologous DNA template is available via a sister chromatid (14). NHEJ does not require a homologous template and is thus not restricted to a certain phase of the cell cycle. A regulatory step which may also play a role in pathway choice is DNA end resection (15). DNA end resection is required for HR-mediated DSB repair and may decrease NHEJ efficiency (15–17). Furthermore, the cell-cycle phase may directly regulate DNA end resection as resection occurs fastest in S phase and CtIP-dependent resection is upregulated by S phase-dependent protein kinases (18–20). Recent studies have implicated Breast Cancer 1, early onset (BRCA1) in playing a role in pathway choice for DSB repair (21–24). BRCA1 is a tumor suppressor that is involved in a multitude of responses to DSBs including playing a role in cell-cycle checkpoint activation, apoptosis and diverse roles in multiple DNA repair pathways (25,26). The BRCA1 protein contains multiple functional domains including an amino-terminal RING domain that has E3 ubiquitin ligase activity and a tandem BRCT domain that facilitates protein–protein interactions via binding to phosphorylated serines (26). BRCA1 plays a multifaceted role in HR as it has been shown to co-localize with a number of HR factors at DSBs, is involved in DNA end resection and facilitates the accumulation of HR factors to damage-induced foci (4,25,26). BRCA1's primary role in the DSB response may be to prevent the accumulation of the pro-NHEJ factors, 53BP1 and Rif1, at DSBs in S phase (23,24). BRCA1 and Rif1/53BP1 mutually antagonize each other, with BRCA1 in conjunction with CtIP promotes the removal of Rif1/53BP1 from DSBs in S phase to allow DNA end resection and Rif1/53BP1 blocks localization of BRCA1 to DSBs in G1(23,24).

Here, we report that BRCA1 attenuates autophosphorylation of DNA-PKcs at the serine 2056 cluster in S phase of the cell cycle. BRCA1 interacts with DNA-PKcs via its tandem BRCT domains in a phospho-independent manner and specifically regulates DNA-PKcs autophosphorylation at the serine 2056 cluster via directly blocking the ability of DNA-PKcs to autophosphorylate itself. Blocking autophosphorylation the DNA-PKcs serine 2056 cluster results in a significant decrease in NHEJ and a subsequent increase in HR-required DNA end processing as monitored by RPA and Rad51 focus formation. We hypothesize that the ability of BRCA1 to attenuate autophosphorylation of DNA-PKcs at the serine 2056 cluster is a mechanism by which cells modulate NHEJ to allow HR-mediated repair in S phase of the cell cycle.

## MATERIALS AND METHODS

### Cell lines, cell culture, transfection, siRNAs and cell synchronization

V3, HeLa and HCC1937 cells were cultured in Hyclone MEM media supplemented with 5% fetal bovine serum and 5% newborn calf serum, 100 U/ml penicillin and 100 U/ml streptomycin at humidified 5% CO_2_ incubation. For HCC1937 cell lines stably expressing FLAG-tagged BRCA1, the cell culture medium was supplemented with 0.4 mg/ml G418. For generation of stable V3 cell lines, cells were transfected using 4.5 μg DNA-PKcs (wild-type or 5A) and 0.5 μg neomycin via an Amaxa Nucleofector kit (Lonza). After selection with 0.5 mg/ml G418 for 14 days, resistant clones were tested for the expression of DNA-PKcs. HCC1937 and HCC1937-BRCA1 cells were transfected using the Amaxa Nucleofector kit with 3 μg DsRed-tagged proliferating cell nuclear antigen (PCNA). For transient transfection and generation of stable cell lines, HeLa cells were transfected with expression plasmids using Lipofectamine 2000 (Invitrogen). Transfection with siRNA oligonucleotides was performed using Lipofectamine RNAi MAX (Invitrogen). All transfections were performed according to the manufacturer's instructions. The following siRNA oligonucleotides were purchased from Thermo Fisher Scientific: GL3 (Luciferase): CUUACGUGAGUACUUCGAUUTT; BRCA1: GGAACCUGUCUCCACAAAGTT. HeLa cell synchronization was carried via a double thymidine block at the G_1_/S boundary and released in normal culture medium for 4 h to obtain S phase and for 12 h to obtain non-S phase cells. Cells were pulse labeled with BrdU for 30 min prior to harvesting and stained with anti-BrdU antibodies for determination of DNA synthesis and with propidium iodide for determination of total DNA content via flow cytometry.

### Antibodies

Commercial antibodies used in this study include: rabbit polyclonal antibodies against BRCA1 (Calbiochem) and Rad51 (Santa Cruz); rabbit monoclonal antibodies against phospho-DNA-PKcs serine 2056 and serine 2612 (Epitomics); mouse monoclonal antibodies against FLAG (Agilent Technologies), phospho-H2AX serine 139 (Millipore), BrdU (Sigma-Aldrich) and RPA2 (Calbiochem); and GST (Santa Cruz), and HisProbe-HRP (Thermo Scientific). In-house made antibodies include mouse monoclonal against DNA-PKcs (25-4) and rabbit polyclonal against phospho-DNA-PKcs serine 2056 and threonine 2609.

### Cloning of FLAG, GST and His_6_-tagged BRCA1 and BRCA1 truncation fragments and GST-tagged DNA-PKcs truncation fragments

Full-length BRCA1 cDNA was amplified using the following polymerase chain reaction (PCR) primers: 5′-GCGGATCCGATTTATCTGCTCTTCGCGTT-3′ (sense strand) and 5′-GCGCTCGAGTCAGTAGTGGCTGTGGGGGAT-3′ (antisense strand). Subsequently, it was cloned into the pCD3F2 vector to add an N-terminal FLAG tag via BamHI and XhoI digestion and ligation. To generate different FLAG-tagged protein fragments of BRCA1, the following digestion scheme was used: amino acids 1–304 was cut by BamHI and EcoRI, residues 302–1863 was cut by EcoRI and XhoI, residues 1313–1863 was cut by BamHI and XhoI and residues 1–1315 was cut by BamHI and these fragments were subcloned into the pCD3F2 vector. The BRCA1 protein fragments encoding amino acids 1–304 and 1313–1863 were also cloned into the pGEX6P vector to generate N-terminal GST-tagged protein fragments. To generate FLAG, His_6_ and GST-tagged BRCA1 protein fragments encoding amino acids 1599–1863, the following PCR primers were used: 5′-CGCGGATCCGCATTGAAAGTTCCCCAATTG-3′ (sense strand) and 5′-GCGCTCGAGTCAGTAGTGGCTGTGGGGGAT-3′ (antisense strand). Subsequently, the PCR product was digested via BamHI and Xho1 and was cloned into the pCD3F2, pQE80 and pGEX6P vectors to add an N-terminal FLAG, His_6_ and GST tag, respectively. To generate GST-tagged DNA-PKcs protein fragments encoding amino acids 1500–2000, 1878–2267 and 2261–2700, the following primers were used: 1500–2000 5′-CCCGGATCCCCTTCTCTAGACCTCAGTTGT-3′ (sense strand) and 5′-GCGCTCGAGTCATCTTTCCATAGGAACCTCAAC-3′ (antisense strand); 1878–2267 5′-GCGGATCCGACGTGATGTATTCTCGCCTTCC-3′ (sense strand) and 5′-GCCTCGAGTCAAGAATTAGGATCTTTACCGGA-3′ (antisense strand); 2261–2700 5′-GCGGATCCTCCGGTAAAGATCCTAATTCT (sense strand) and 5′-GCAAGCTTGAATTCTCAAAAATCAGGCCCCACTGAC-3′ (antisense strand). Subsequently, the PCR product encoding amino acids 1500–2000 was digested with BamHI, 1878–2267 with EcoRI and 2261–2700 with HindIII and cloned into the pGEX6P vector to add an N-terminal GST tag. All the sequences were verified by automated sequencing.

### Purification of GST- and His_6_-tagged BRCA1 fragments, GST-tagged fragments of DNA-PKcs and XRCC4 and endogenous full-length DNA-PKcs

GST-tagged BRCA1 fragments (amino acids 1–304; 1313–1863 and 1599–1863) and GST-tagged DNA-PKcs fragments (amino acids 1500–2000; 1878–2182 and 2261–2700) were expressed in bacteria and purified as previously described (27). The purified GST-tagged BRCA1 and DNA-PKcs fragments were either left attached to the glutathione sepharose (GE Healthsciences) or eluted using 10 mM fresh reduced glutathione in 50 mM Tris-HCl, pH 8.0. The eluted protein was dialyzed against Buffer C (20 mM HEPES, pH 7.5, 100 mM KCl, 1 mM DTT, 1 mM ethylenediaminetetraacetic acid (EDTA) and 20% glycerol) and stored at −80ºC until used. His_6_-tagged BRCA fragment (amino acids 1599–1863) was expressed in bacteria and purified using Ni-NTA (Qiagen) under native conditions as outlined in the manufacturer's manual. Following elution and dialyzing against Buffer C, the protein was stored at −80ºC until used. GST fusion XRCC4 C-terminal fragments were prepared as previously described (28). Endogenous full-length DNA-PKcs was purified as described recently (29).

### Immunoprecipitations and *in vitro* protein–protein interaction assays

For immunoprecipitations, HeLa cells were irradiated with 10 Gy of irradiation (IR) or mock irradiated and allowed to recover for 60 min. The cells were washed with cold phosphate buffered saline (PBS) and then lysed using Lysis buffer (50 mM Tris pH7.5, 250 mM NaCl, 1 mM EDTA, 0.1% NP-40, 1 mM phenylmethylsulfonyl sulfate (PMSF) and protease inhibitors) on ice for 20 min. Lysates were cleared by centrifugation at 14,000 revolutions per minute at 4°C for 20 min and supernatants were incubated with protein G agarose (Roche) conjugated with primary antibodies at 4°C overnight with mixing. The immunocomplexes were washed three times with Lysis buffer and subjected to sodium dodecyl sulphate-polyacrylamide gel electrophoresis (SDS-PAGE) and western blotting analysis using the antibodies specified in the figure legends. For *in vitro* interaction assays, HeLa cells were irradiated with 20 Gy of IR or mock irradiated and DNA-PKcs was immunoprecipitated (IPed) utilizing the DNA-PKcs antibody 25-4 and protein A sepharose with mixing for 2 h at 4ºC. The protein A beads were washed three times with wash buffer (50 mM Tris pH 7.5, 150 mM NaCl, 1 mM EDTA, 1% NP-40, 10% glycerol and protease inhibitors), and untreated or treated with 1200 U of λ-phosphatase (New England Biolabs) for 30 min at 30ºC and then incubated with purified GST or GST-tagged BRCA1 fragments in wash buffer supplemented with 10 μg bovine serum albumin (BSA) at ambient temperatures for 2 h with mixing. After washing three times with wash buffer, bound proteins were solubilized in SDS-PAGE loading buffer and analyzed by Western blotting using the antibodies indicated in the figure legends. For the *in vitro* interaction reactions with N-PKcs and C-PKcs, FLAG-tagged N-PKcs or C-PKcs was IPed from Sf9 lysates using FLAG antibody and protein A sepharose. The FLAG precipitates were washed and incubated with GST or GST-tagged fragments of BRCA1 in wash buffer supplemented with BSA and the assay was performed as described above. *In vitro* interaction assays with fragments of DNA-PKcs were performed using purified GST-tagged fragments of DNA-PKcs still bound to the glutathione sepharose and incubated with purified His_6_-tagged BRCA1 tandem BRCT domain and the assay was completed as described above.

### *In vitro* kinase assays and verification of DNA-PKcs phosphorylation *in vivo*

*In vitro* kinase assay were performed as previously described (29). The kinase reactions were performed in the presence or absence of GST or GST-tagged tandem BRCT domain of BRCA1 with the final volume of the reaction being either 10 or 20 μl. The reactions were incubated for 30 min at 30ºC, terminated by the addition of SDS-PAGE sample buffer, resolved via a 12% SDS-PAGE and γ-^32^P incorporation was detected by PhosphoImager analysis (Amersham Biosciences). HeLa cells which were or were not stably expressing BRCT were mock or irradiated with 10 Gy of IR and allowed to recover for 30 min. Nuclear extracts were isolated and Western blot analysis was performed using the antibodies indicated in the figure legend.

### Laser micro-IR

A 365-nm pulsed nitrogen laser (Spectra-Physics) was directly coupled to the epiflourescence path of the microscope (Axiovert 200M; Carl Zeiss) and used to generate DSBs in a defined area of the nucleus as described previously (30).

### Fluorescent immunostaining

For immunostaining, cells were grown on coverslips and irradiated via the 365-nm pulsed nitrogen laser (Spectra-Physics) or 8 Gy of IR. For the cells irradiated with the laser, the coverslip was placed in a dark box and the cells were allowed to recover at 37°C for 30 min. The cells were subsequently washed with ice cold PBS three times and then fixed via incubation with 4% paraformaldehyde at room temperature for 20 min. The cells were permeablized with PBS containing 0.5% Triton X-100 on ice for 10 min, blocked with PBS containing 5% normal goat serum overnight at 4°C and then incubated with the primary antibodies at room temperature for 1 h. After washing with cold PBS, cells were incubated with the second antibodies conjugated with Alexa 488 (Invitrogen, A11001) or Texas red (Invitrogen, T2767). Following IR with 8 Gy, focus formation of RPA and Rad51 was detected using fluorescent immunostaining at various time points as described in the figure legends and as previously described (30).

### Cell survival

Survival curves for each cell line were obtained by measuring the colony-forming abilities of irradiated cell populations. Cells were irradiated at the doses of 1, 2, 3, 4 or 6 Gy and then plated on 60-mm plastic Petri dishes. After 7 days, cells were fixed with 100% ethanol and stained with 0.1% crystal violet in a 100% ethanol solution, colonies containing more than 50 cells were scored and the mean value for triplicate culture dishes was determined. Cell survival was normalized to plating efficiency of untreated controls for each cell type.

## RESULTS

### DNA-PKcs localizes to DSBs but its autophosphorylation at serine 2056 is attenuated in S phase of the cell cycle

**Figure 1.**
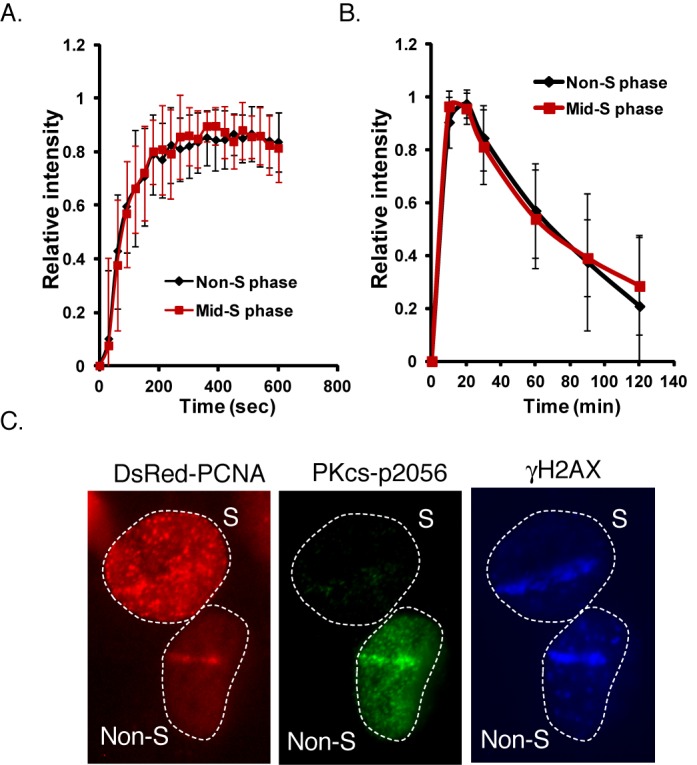
Dynamics of DNA-PKcs at DSBs is not cell-cycle regulated but autophosphorylation at serine 2056 is attenuated in S phase. YFP-tagged DNA-PKcs was transiently expressed in U2-OS cells with DsRed-tagged PCNA to differentiate S and non-S phase cells. Initial localization **(A)** and kinetics **(B)** of relative fluorescent intensity of YFP-tagged DNA-PKcs in U2-OS at DSBs after micro-IR in S and non-S phase cells. **(C)** Laser-generated DSBs were produced in U2-OS cells transiently expressing DsRed-tagged PCNA to differentiate S and non-S phase cells followed by immunofluorescence assays utilizing phospho-DNA-PKcs serine 2056 and γH2AX antibodies to probe DNA-PKcs autophosphorylation and DSBs, respectively.

The cell-cycle phase is believed to play a significant role in the balance between HR and NHEJ for the repair of specific breaks, with HR being the prominent repair pathway in S and G2 phases of the cell cycle. A possible mechanism allowing HR to occur instead of NHEJ is that the HR machinery outcompetes the NHEJ machinery for the DSB in S/G2 phases. However, our previous findings showed that Ku70/80 localizes to DSBs in S phase similar to non-S phase implicating that the competition model may not be correct (30). To expand on this previous result, we examined if recruitment of other canonical NHEJ factors to DSBs is attenuated in S phase. We first assessed the ability of YFP-tagged DNA-PKcs to localize to laser-generated DSBs via micro-IR using a 365 nm laser in U2-OS cells in S and non-S phase cells. Transiently expressed DsRed-tagged PCNA was utilized to differentiate S and non-S phase cells as PCNA forms distinct foci in S phase, whereas it shows faint and even expression in non-S phase cells (30,31). Comparable to the Ku heterodimer, DNA-PKcs rapidly localizes to laser-generated DSBs and time-lapse imaging showed that the initial recruitment of DNA-PKcs is similar in S and non-S phase cells (Figure 1A). Time-lapse imaging showed that the kinetics of DNA-PKcs in S and non-S phase cells are similar with the fluorescence intensity peaking at roughly 15 min and 80% of the protein dissociated from DSBs after 120 min (Figure 1B). Furthermore, the initial localization of XRCC4 to laser-generated DSBs is the same in S and non-S phase cells (data not shown). Collectively, the data show the canonical NHEJ factors localize to DSBs in S phase and suggests that direct competition between HR and NHEJ factors for DSBs is likely not responsible for attenuation of NHEJ in S phase.

Since the canonical NHEJ factors localize to DSBs in S phase, we speculated that there is a mechanism(s) that attenuates NHEJ for HR to occur in S phase. A long standing observation is that autophosphorylation of DNA-PKcs at serine 2056 is attenuated in S phase and thus is a potential S phase-specific mechanism responsible for modulating NHEJ (10). To verify that DSB-induced DNA-PKcs autophosphorylation at serine 2056 is attenuated in S phase, cells transiently expressing DsRed-tagged PCNA to differentiate S and non-S phase cells were microirradiated and immunofluorescence assays were performed using a phospho-specific antibody to serine 2056. The immunofluorescence assays show that autophosphorylation of DNA-PKcs at serine 2056 is attenuated in S phase compared to non-S phase cells (Figure 1C). γH2AX, an established marker for DSBs, was detected along the micro-IR track in both S and non-S phase cells verifying that DSBs were generated. Furthermore, we found that IR-induced phospho-2056 focus formation is attenuated in S phase (data not shown). These data verify the previous published data that autophosphorylation of DNA-PKcs at serine 2056 is attenuated in S phase of the cell cycle and spurred us to further explore the role that attenuating autophosphorylation of DNA-PKcs at serine 2056 plays in attenuating NHEJ specifically in S phase.

### BRCA1 attenuates autophosphorylation of DNA-PKcs at serine 2056 in S phase of the cell cycle

**Figure 2.**
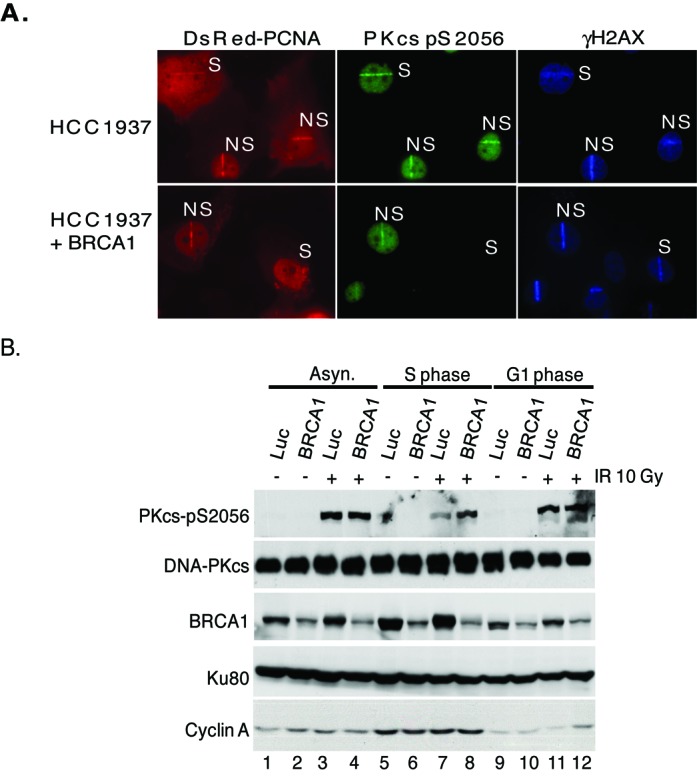
BRCA1 attenuates autophosphorylation of DNA-PKcs at serine 2056 in S phase. **(A)** BRCA1 defective HCC1937 cells were uncomplemented or complemented with FLAG-tagged BRCA1. Using DsRed-tagged PCNA to differentiate S and non-S phase cells, laser-generated DSBs were produced in the cells followed by immunofluorescence using phospho-DNA-PKcs serine 2056 and γH2AX antibodies to probe DNA-PKcs autophosphorylation and DSB formation, respectively. **(B)** Exponentially growing HeLa cells were transfected with luciferase control (Luc) or BRCA1 siRNA and then either left asynchronous (Asyn.) or synchronized in G1 or S phase of the cell cycle. The cells were either mock or irradiated with 10 Gy and allowed to recover for 1 h. Whole cell lysates were prepared and analyzed by western blot analysis for DNA-PKcs, phosphorylated DNA-PKcs at serine 2056, BRCA1, Ku80 and cyclin A.

Next, we aimed to identify a factor responsible for modulating autophosphorylation of DNA-PKcs at serine 2056 in S phase. We hypothesized that BRCA1 may be the responsible factor as it functions primarily in S phase, has been implicated in DSB repair pathway choice and has been found to interact with the DNA-PK factor, Ku80 (21–24,32,33). Using DsRed-tagged PCNA to differentiate the cell-cycle stage, autophosphorylation of DNA-PKcs was assessed following laser-generated DNA damage in the BRCA1 defective cell line HCC1937 and HCC1937 cells stably expressing FLAG-tagged BRCA1 in S and non-S phase. The immunofluorescence assays show that DNA-PKcs was clearly autophosphorylated at serine 2056 in HCC1937 cells and HCC1937 cells complemented with BRCA1 in non-S phase cells (Figure 2A). In S phase cells, DNA-PKcs was autophosphorylated at serine 2056 in HCC1937 cells but autophosphorylation of DNA-PKcs was attenuated in the HCC1937 cells complemented with BRCA1, suggesting BRCA1 regulates DNA-PKcs serine 2056 phosphorylation (Figure 2A).

To support this data, HeLa cells were treated with non-specific control siRNAs or with specific siRNAs to knock-down the protein level of BRCA1, then either left cycling or synchronized in S phase or non-S phase (population consisting of cells mostly in G1 and the rest in G2), mock irradiated or irradiated with 10 Gy and finally nuclear extracts were produced to assess the phosphorylation status of DNA-PKcs at serine 2056. The synchronization of the cells was assessed via flow cytometry (data not shown) and expression of cyclin A, which is upregulated in S phase, was used as a western blot control (Figure 2B). As previously shown, DNA-PKcs autophosphorylation at serine 2056 is attenuated in S phase cells compared to non-S and asynchronous cells (∼60% decrease in S phase compared to non-S phase) (Figure 2B, compare lanes 3, 7 and 11). Knock-down of BRCA1 did not affect the autophosphorylation status of DNA-PKcs in non-S or asynchronous cells, but did result in a marked increase in S phase cells (∼90% increase in S phase compared to non-S phase) (Figure 2B, compare lanes 3, 4, 7, 8, 11 and 12). Collectively, the data implicate that BRCA1 plays a role in regulating the phosphorylation status of DNA-PKcs at the serine 2056 cluster in S phase of the cell cycle.

### BRCA1-DNA-PKcs interaction is cell cycle regulated

**Figure 3.**
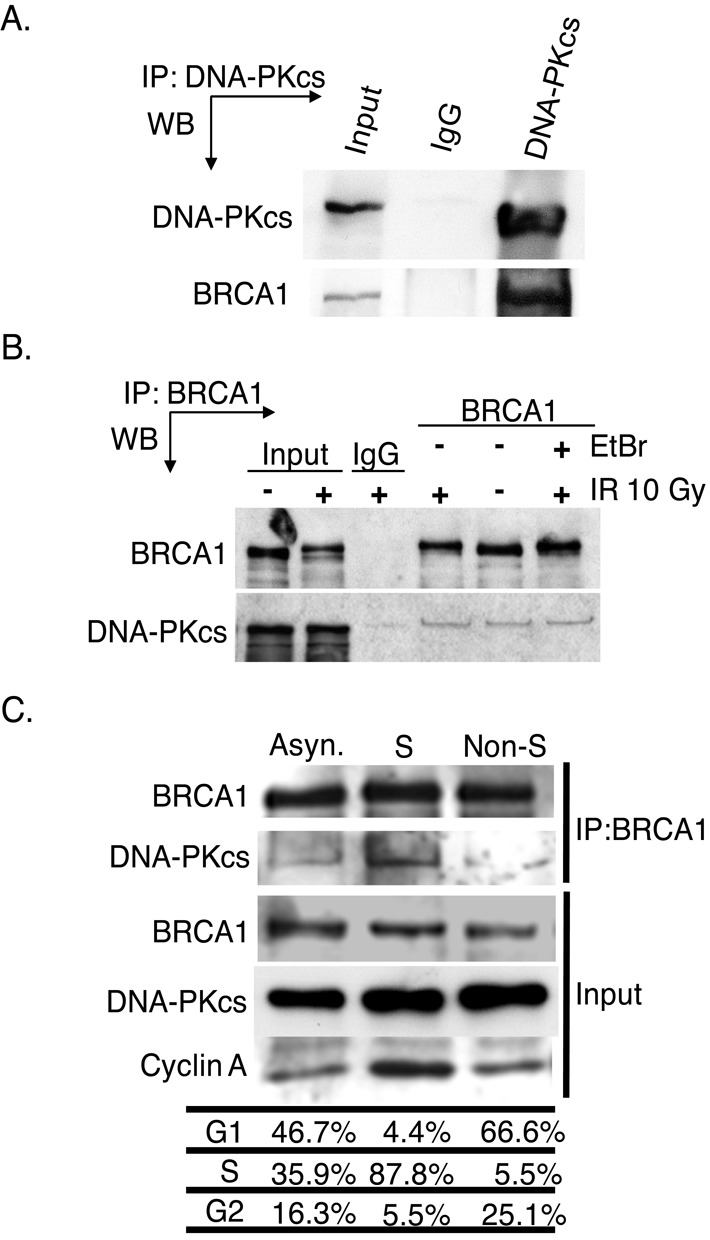
DNA-PKcs interacts with BRCA1 independently of DNA damage but is specific for S phase of the cell cycle. **(A)** DNA-PKcs was IPed from exponentially growing HeLa cells using an antiserum specific for DNA-PKcs or non-specific mouse immunoglobulin G (IgG). The immunoprecipitates were analyzed by western blot (WB) analysis using anti-DNA-PKcs or anti-BRCA1 antibodies. Input is 10% of total. (**B**) Exponentially growing HeLa cells were non-irradiated or irradiated with 10 Gy and allowed to recover for 1 h. Subsequently, BRCA1 was IP from HeLa cells using antiserum against BRCA1 or non-specific rabbit IgG. Furthermore, 100 μg/ml ethidium was or was not added to the irradiated lysate to disrupt non-specific DNA-protein interactions. Western blot analysis was then performed using antibodies against DNA-PKcs or BRCA1. Input is 10% of total. **(C)** HeLa cells were left asynchronized (Asyn.) or synchronized and released and harvested when the population was primarily in S or non-S phase followed by immunoprecipitation of BRCA1. Western blot analysis was then performed using antibodies against BRCA1 and DNA-PKcs to assess their interaction and cyclin A to monitor S phase.

Since the presence of BRCA1 is required for the decrease in DNA-PKcs autophosphorylation at serine 2056 in S phase, we next determined if this was direct by first testing if BRCA1 and DNA-PKcs associate. Immunoprecipitation assays show that endogenous BRCA1 or DNA-PKcs associate with each other in reciprocal immunoprecipitation assays (Figure 3A and B). The association between BRCA1 and DNA-PKcs was not DNA damage responsive as treating cells with 10 Gy of γ-IR did not affect the interaction (Figure 3B). Furthermore, the association between BRCA1 and DNA-PKcs, two known DNA interacting proteins (34,35), is not mediated by non-specific DNA-protein interactions as the addition of ethidium bromide did not disrupt the BRCA1-DNA-PKcs association (Figure 3B). Next, it was examined if the association between BRCA1 and DNA-PKcs is cell-cycle regulated. HeLa cells were left cycling or synchronized in S and non-S phase and BRCA1 was IPed. The association between BRCA1 and DNA-PKcs increased in the cell population that were in S phase compared to non-S phase cells (Figure 3C). Analysis found ∼82% decrease in the association between BRCA1 and DNA-PKcs in non-S phase compared to S phase. Together, the data clearly show that BRCA1 associates with DNA-PKcs independently of DNA damage, but in a cell cycle-dependent manner.

### DNA-PKcs interacts with the C-terminus of BRCA1

**Figure 4.**
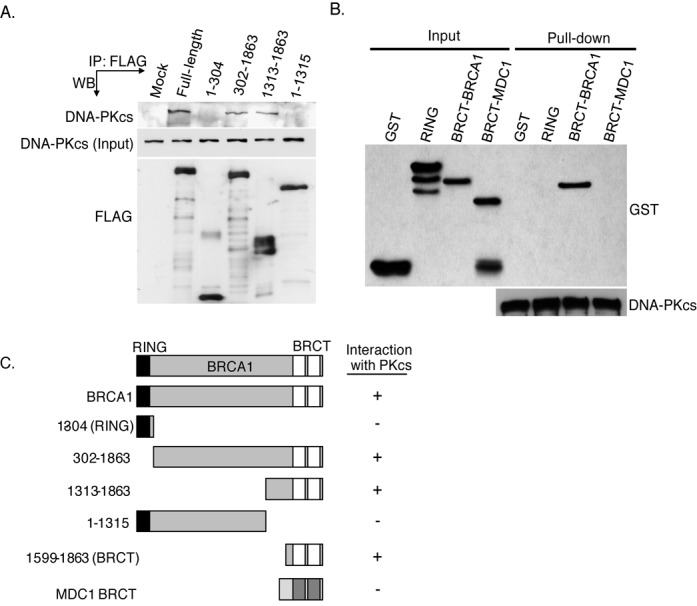
DNA-PKcs interacts with the tandem BRCT domain of BRCA1. **(A)** FLAG-tagged fragments of BRCA1 were transiently expressed in HeLa cells and subsequently IPed using an antiserum specific for the FLAG-tag. The immunoprecipitates were analyzed by western blot analysis using anti-DNA-PKcs or anti-FLAG antibodies. **(B)** DNA-PKcs was IP from HeLa cells, washed three times and the IP DNA-PKcs which was still bound to the protein A-sepharose was incubated with purified GST or GST-tagged tandem BRCT domain of BRCA1 or MDC1. The pull-downs were then washed and western blot analysis was performed using antibodies against GST or DNA-PKcs. **(C)** Representation of BRCA1 fragments used in (D and E) and their ability to interact with DNA-PKcs.

As DNA-PKcs and BRCA1 associate, we next aimed to further characterize and identify the motifs/residues mediating this protein–protein interaction. To identify which region of BRCA1 associates with DNA-PKcs in intact cells, FLAG-tagged BRCA1 protein fragments were transiently expressed and IPed from HeLa cells and it was examined which BRCA1 fragment interacted with endogenous DNA-PKcs. DNA-PKcs co-precipitated with FLAG-tagged full-length BRCA1 (Figure 4A) and the association was not affected by IR (Supplementary Figure S1). The DNA-PK complex factor Ku80 has been reported to interact with BRCA1, with one study showing amino acids 1–304 and the other reporting amino acids 262–803 of BRCA1 are required for the BRCA1-Ku80 interaction (32,33). DNA-PKcs did not associate with the BRCA1 protein fragments spanning amino acids 1–304 or 1–1315 suggesting that DNA-PKcs associates with BRCA1 independently of Ku80 (Figure 4A and C). DNA-PKcs was found to associate with the BRCA1 fragments spanning amino acids 302–1863 and 1313–1863 implicating that DNA-PKcs specifically interacts with the C-terminal region of BRCA1 (Figure 4A and C). To further delineate the region of BRCA1 which DNA-PKcs interacts with, GST-tagged fragments of the C-terminal region of BRCA1 were used in *in vitro* interaction assays with IPed DNA-PKcs. Similar to the immunoprecipitation assays, GST-tagged RING finger domain of BRCA1 (amino acids 1–304, termed ‘RING’) did not interact with DNA-PKcs *in vitro*, but a fragment of BRCA1 which encodes only the tandem BRCT domain of BRCA1 (termed ‘BRCT’) did (Figure 4B and C). Furthermore, this interaction is specific for the tandem BRCT domain of BRCA1 as DNA-PKcs does not associate with the tandem BRCT domain of MDC1 (Figure 4B and C). Collectively, the data show that DNA-PKcs interacts with the tandem BRCT domain of BRCA1, which is located in the C-terminus of the protein and the interaction between DNA-PKcs and BRCA1 is different than that between BRCA1 and Ku80.

### The interaction between DNA-PKcs and the tandem BRCT domain of BRCA1 is phospho-independent

**Figure 5.**
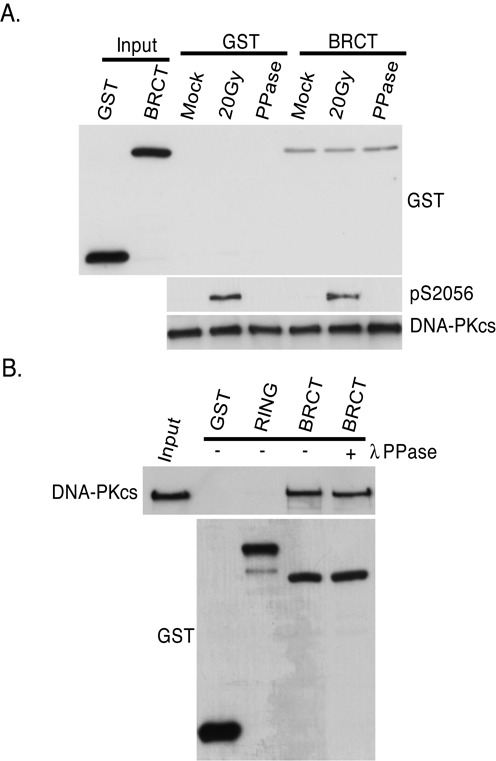
The interaction between DNA-PKcs and the tandem BRCT domain of BRCA1 is phospho-independent. **(A)** Exponentially HeLa cells were mock or irradiated with 20 Gy and allowed to recover for 1 h. DNA-PKcs was IPed, washed three times and the IP DNA-PKcs which was still bound to the protein A-sepharose was incubated with GST or GST-tagged BRCA1 tandem BRCT domain (BRCT) protein fragment in the presence or absence of lambda phosphatase (PPase). The pull-downs were then washed and western blot analysis was performed using antibodies against GST or DNA-PKcs. Antibodies against phosphorylated serine 2056 were used to show IR-induced phosphorylation of DNA-PKcs. **(B)** Purified DNA-PKcs was incubated with GST or GST-tagged protein fragments of BRCA1 encoding the RING finger domain (RING) or the tandem BRCT domain (BRCT) in the presence or absence of lambda phosphatase (PPase). The pull-downs were then washed and western blot analysis was performed using antibodies against GST or DNA-PKcs.

The tandem BRCT domain of BRCA1 mediates a number of protein–protein interactions via specifically associating with proteins phosphorylated on serine residues (36). As DNA-PKcs is a heavily phosphorylated protein, it was assessed if BRCA1 associates with DNA-PKcs in a phospho-dependent manner utilizing *in vitro* binding assays similar to those described above. HeLa cells were either mock irradiated or irradiated with 20 Gy of IR to stimulate IR-induced phosphorylation of DNA-PKcs followed by immunoprecipitation of DNA-PKcs. Subsequently, the mock-irradiated IPed DNA-PKcs was or was not treated with alkaline phosphatase to dephosphorylate any non-DNA damage-inducible DNA-PKcs phosphorylations and then incubated with either GST or BRCT. IR-induced phosphorylation of DNA-PKcs at serine 2056 (Figure 5A) and threonine 2609 and serine 2612 (data not shown) were observed via Western blot analysis, whereas there was no detectable phosphorylation at any of these three residues in the phosphatase treated sample. The *in vitro* interaction assays showed that neither IR-induced phosphorylation nor phosphatase treatment of DNA-PKcs affected its interaction with BRCT (Figure 5A). Next, *in vitro* GST-pull-down assays were performed with GST, RING and BRCT and purified endogenous DNA-PKcs that was or was not treated with alkaline phosphatase to remove any phosphorylations of DNA-PKcs which may have occurred in normal cycling cells or during purification. DNA-PKcs specifically interacted with BRCT but not GST or RING and this interaction was not affected by phosphatase treatment of DNA-PKcs (Figure 5B). Collectively, the data illustrate that DNA-PKcs interacts with the tandem BRCT domain of BRCA1 and does so in a phospho-independent manner.

### BRCA1 interacts with the N-terminal region of DNA-PKcs near the serine 2056 cluster

**Figure 6.**
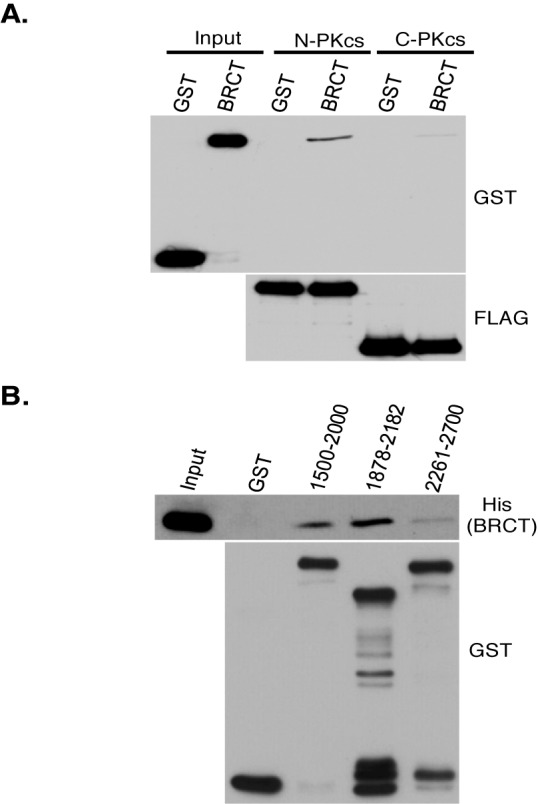
BRCA1 preferentially interacts with the N-terminal region of DNA-PKcs near the 2056 cluster. **(A)** FLAG-tagged N-terminal fragment (N-PKcs) and C-terminal fragment (C-PKcs) of DNA-PKcs were expressed and IPed from Sf9 cells using FLAG antibody, washed three times and the IP DNA-PKcs fragments still bound to the protein A-sepharose were incubated with GST or BRCT. Western blot analysis was then performed using antibodies against GST or FLAG. **(B)** GST-tagged fragments of DNA-PKcs encoding the amino acids indicated in the figure were purified from bacteria and left bound on the glutathione-agarose. These fragments were then incubated with purified His-tagged BRCA1 tandem BRCT domain protein fragment (BRCT). Following the incubation, the GST pull-downs were washed and western blot analysis was performed using anti-His and GST antibodies.

To further characterize the BRCA1-DNA-PKcs interaction it was next determined which region of DNA-PKcs mediates its interaction with BRCA1. Utilizing two large and distinct fragments of DNA-PKcs corresponding to amino acids 1–2713 (termed N-PKcs) and amino acids 2714–4128 (termed C-PKcs), *in vitro* interaction assays were performed to first assess which region of BRCA1 interacts with DNA-PKcs (29). FLAG-tagged N-PKcs or C-PKcs were expressed and IPed from Sf9 cells and then incubated with either purified GST or BRCT. The *in vitro* interaction assays show that the BRCT preferentially interacts with N-PKcs (Figure 6A). GST pull-downs were then performed utilizing different GST-tagged protein fragments of the N-terminal region of DNA-PKcs and His-tagged BRCT to identify specific residues of DNA-PKcs mediating its interaction with BRCA1. His-tagged BRCT weakly interacts with a fragment of DNA-PKcs encoding amino acids 1500–2000 but more strongly interacts with a fragment encoding 1878–2182 (Figure 6B). However, His-tagged BRCT's interaction with the DNA-PKcs fragment encoding amino acid 2261–2700 was only slightly above background levels (Figure 6B). These data implicate that BRCA1 interacts with the region at and surrounding the serine 2056 cluster, but not the region at the threonine 2609 cluster.

### BRCA1 attenuates autophosphorylation of DNA-PKcs at serine 2056 by directly blocking phosphorylation of this site *in vitro* and *in vivo*

As BRCA1 plays a role in regulating the phosphorylation status of DNA-PKcs at serine 2056 in S phase of the cell cycle, we next aimed to identify the mechanism by which BRCA1 modulates DNA-PKcs autophosphorylation. First, it was assessed if the observed decrease in DNA-PKcs autophosphorylation in BRCA1 proficient cells in S phase was due to the inability of DNA-PKcs to localize to or be retained at DSBs. U2-OS cells were treated with non-specific control siRNAs or with specific siRNAs to knock-down the protein level of BRCA1 and the ability of YFP-tagged DNA-PKcs to localize to DSBs was assessed in S and non-S phase cells. YFP-tagged DNA-PKcs quickly localized to the laser-generated DSBs and its overall dynamics were similar in S and non-S phase cells, which were treated with either non-specific siRNAs or BRCA1 siRNAs (Supplementary Figure S2). The data clearly show that BRCA1 does not play a role in regulating the ability of DNA-PKcs to localize to or dissociate from DSBs.

**Figure 7.**
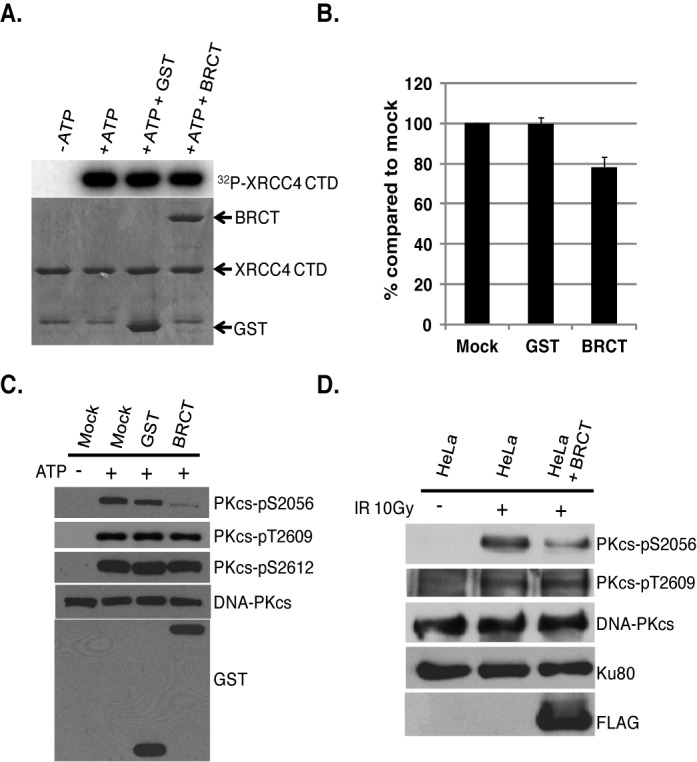
The tandem BRCT domains of BRCA1 attenuate autophosphorylation of DNA-PKcs at serine 2056 *in vitro* and *in vivo. In vitro* kinase assays were performed to assess DNA-PKcs-mediated phosphorylation of XRCC4 CTD in the absence or the presence of GST or BRCT. **(A)** Incorporation of ^32^P-ATP into GST-XRCC4 CTD as measured by PhosphoImager analysis. **(B)** The incorporation ^32^P-ATP into GST-XRCC4 CTD was calculated using PhosphoImager software and % was compared to mock. **(C)** *In vitro* DNA-PKcs kinase assays were performed in the absence or presence of purified GST or GST-tagged BRCA1 tandem BRCT domain protein fragment (BRCT). Western blot analysis was performed to test *in vitro* autophosphorylation of DNA-PKcs using phospho-antibodies against phosphorylated serine 2056, threonine 2609 and serine 2612. Antibodies against DNA-PKcs and GST were used to show protein amounts of DNA-PKcs and the GST-tagged proteins in the assay. **(D)** IR-induced phosphorylation of DNA-PKcs at serine 2056 and threonine 2609 was compared in HeLa cells stably expressing FLAG-tagged BRCA1 tandem BRCT domain protein fragment (BRCT) with untransfected cells. The exponentially growing HeLa cells were either mock or irradiated with 10 Gy and allowed to recover for 1 h and whole cell lysates were analyzed by western blot analysis for DNA-PKcs, phosphorylated DNA-PKcs at serine 2056 and threonine 2609, FLAG and Ku80 (loading control).

It was next assessed if BRCA1 attenuates DNA-PKcs autophosphorylation at the serine 2056 cluster via modulation of the overall kinase activity of DNA-PKcs. Previous studies have shown that DNA-PKcs kinase activity is inhibited in cell-free kinase assays by the monoclonal antibody 18-2, which interacts with amino acids 2001–2025 of DNA-PKcs (37,38). As BRCA1 interacts with DNA-PKcs near the 18-2 epitope, it is possible that BRCA1 may directly regulate DNA-PKcs autophosphorylation by blocking its overall kinase activity. To test this, *in vitro* kinase assays were performed in the presence of GST or BRCT and the ability of DNA-PKcs to phosphorylate the carboxy-terminal domain (CTD) of XRCC4 was assessed. Addition of purified GST did not affect the overall kinase activity of DNA-PKcs (Figure 7A). The BRCA1 tandem BRCT domain protein fragment inhibited overall kinase activity of DNA-PKcs by ∼20% (Figure 7A and B) suggesting that BRCA1 binding to DNA-PKcs may affect its overall kinase activity, albeit moderately.

Since the tandem BRCT domain interacts with DNA-PKcs in the region near or at the serine 2056 cluster, it is also possible that BRCA1 may directly block the ability of DNA-PKcs to autophosphorylate itself at this cluster. To test this, *in vitro* kinase assays were performed in the presence of GST or BRCT and autophosphorylation at serine 2056, threonine 2609 and serine 2612 was assessed. Addition of purified GST slightly affected autophosphorylation at serine 2056 (∼20% decrease compared to autophosphorylation in the absence of GST and BRCT), but addition of BRCT resulted in a marked decrease in autophosphorylation specifically at serine 2056 (∼85% decrease) (Figure 7C). GST or BRCT did not affect autophosphorylation at threonine 2609 or serine 2612. Next, HeLa cells stably expressing a FLAG-tagged BRCA1 BRCT domain protein fragment were created and it was determined if overexpression of this protein fragment of BRCA1 attenuated IR-induced autophosphorylation of DNA-PKcs at serine 2056 *in vivo*. As seen in Figure 7D, HeLa cells expressing FLAG-tagged BRCT show a significant decrease (∼55% decrease compared to cells without the BRCA1 protein fragment) in IR-induced phosphorylation of DNA-PKcs at serine 2056 but not threonine 2609 *in vivo*. Taken together, the BRCA1 tandem BRCT domain protein fragment blocks phosphorylation of DNA-PKcs at serine 2056 but not threonine 2609 or serine 2612 *in vitro* and *in vivo*. Although binding of BRCT to DNA-PKcs modestly inhibited DNA-PKcs kinase activity, we surmise the ability of BRCA1 to regulate autophosphorylation of DNA-PKcs at the serine 2056 phosphorylation cluster is likely via its ability to directly occlude the autophosphorylation site.

### Blocking phosphorylation of DNA-PKcs at the serine 2056 cluster results in radiosensitivity and an increase in the DNA end processing required for HR-mediated repair

The data presented above suggests that BRCA1 interacts directly with the region of DNA-PKcs near or at the serine 2056 cluster and blocks phosphorylation of this cluster in S phase of the cell cycle. Next, we wanted to determine if the phosphorylation status of DNA-PKcs at the serine 2056 cluster attenuates NHEJ and subsequently results in an increase in the DNA end processing required for HR-mediated repair. Previous studies showed that expression of alanine-substituted DNA-PKcs at serine 2056 and the serine 2056 cluster in the DNA-PKcs deficient CHO (V3) cell line caused radiosensitivity compared with V3 cells complemented with wild-type DNA-PKcs (12,39,40). First, new V3 cell lines stably expressing wild-type DNA-PKcs (WT) or DNA-PKcs in which phosphorylation at the serine 2056 phosphorylation cluster (5A) was ablated were isolated. Cells expressing DNA-PKcs in which the entire 2056 phosphorylation cluster was ablated were utilized as a previous study showed that phosphorylation of one or two sites within the cluster could suffice functionally and thus full ablation of the cluster is required (12). The uncomplemented V3 cells had undetectable levels of DNA-PKcs, whereas the V3-WT and V3-5A cells showed similar levels of expression of the DNA-PKcs protein (Supplementary Figure S3A). Similar to previous results, cells complemented with 5A were significantly radiosensitive compared to V3 cells complemented with wild-type DNA-PKcs, but not as radiosensitive as uncomplemented V3 cells (Supplementary Figure S3B) (12,40). The data show that autophosphorylation of DNA-PKcs at the 2056 cluster is required for resistance to ionizing radiation and is critical for the repair of DSBs by the NHEJ pathway.

**Figure 8.**
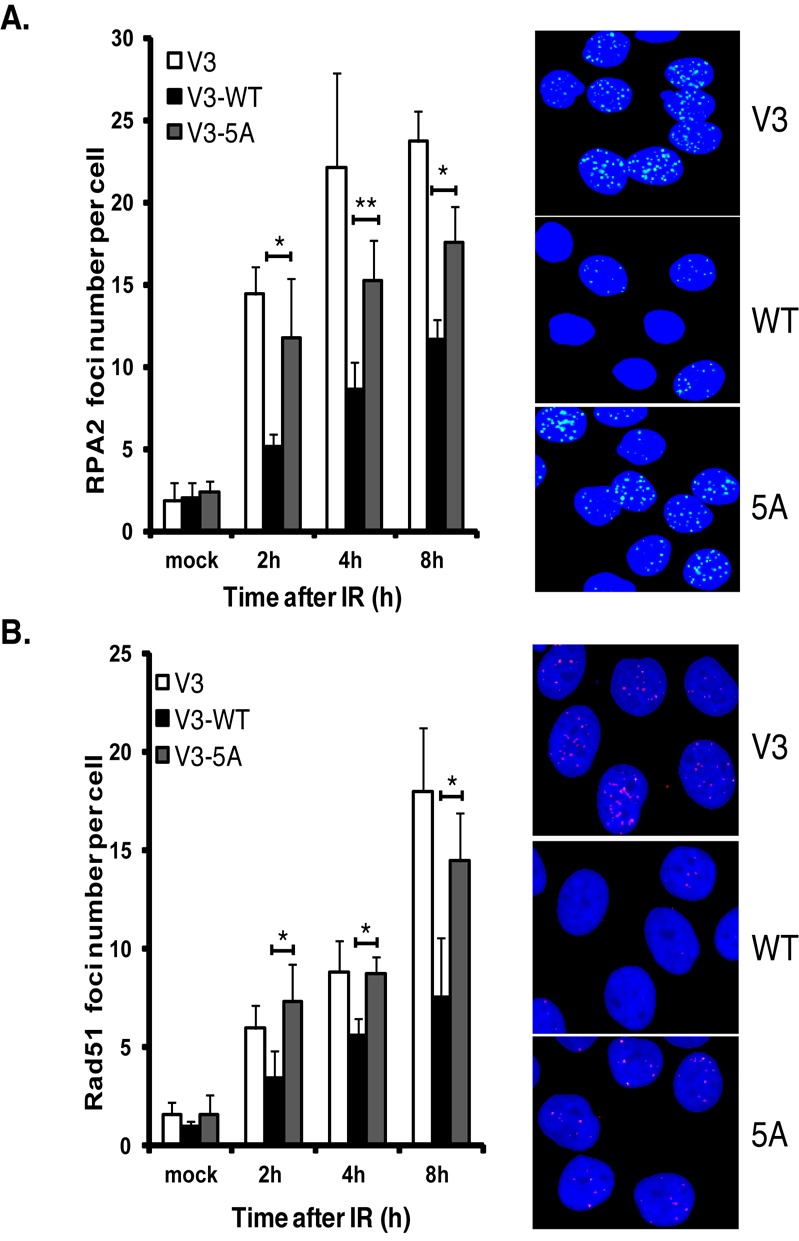
Blocking phosphorylation of DNA-PKcs at the serine 2056 cluster results in an increase in DNA end processing required for HR. **(A)** RPA foci formation following DNA damage is increased in DNA-PKcs deficient (V3) cells complemented with DNA-PKcs in which the serine 2056 cluster is ablated (5A) compared to wild-type complemented cells. Immunostaining of V3 or V3 complemented with wild-type or 5A DNA-PKcs after 8 Gy of γ-ray. Cells were pre-extracted and fixed 1, 2 or 4 h after IR and immunostained for RPA. RPA foci were counted per cell and averaged. Student's *t*-test was performed to test significance. *< 0.05; **< 0.01. **(B)** Rad51 foci formation following DNA damage is increased in DNA-PKcs deficient (V3) cells complemented with DNA-PKcs in which the serine 2056 cluster is ablated (5A) compared to wild-type complemented cells. Immunostaining of V3 or V3 complemented with wild-type or 5A DNA-PKcs after 8 Gy of γ-ray. Cells were pre-extracted and fixed 2, 4 or 8 h after IR and immunostained for Rad51. Rad51 foci were counted per cell and averaged. Student's *t*-test was performed to test significance. *< 0.05; **< 0.01. Each image in (A) and (B) was taken at the 8-h time point.

Since blocking phosphorylation at the serine 2056 cluster results in a decrease in NHEJ, it was next assessed if blocking phosphorylation of these sites results in an increase in DNA end resection and recruitment of HR proteins to IR-induced DSBs. As DNA end resection generates ssDNA ends that are rapidly coated by RPA, RPA focus formation following DNA damage is utilized as a surrogate marker for DNA end resection. To first determine if blocking phosphorylation of DNA-PKcs at the serine 2056 cluster results in an increase in DNA end resection, RPA focus formation was assessed in V3 cells either uncomplemented or complemented with WT or 5A. Expression of 5A results in a marked and significant increase in the number of RPA foci per cells (Figure 8A) and the percent of cells with >10 RPA foci (Supplementary Figure S4A) at 2, 4 and 8 h post-IR compared to V3 cells expressing wild-type DNA-PKcs. To further test if blocking phosphorylation of DNA-PKcs at the serine 2056 cluster results in an increase in the early steps of HR, it was next assessed if Rad51 focus formation was increased in cells expressing 5A compared to wild-type DNA-PKcs. Rad51 replaces RPA on the ssDNA and is required for strand invasion for HR to occur and thus Rad51 focus formation is an established marker for ongoing HR-mediated DSB repair. Expression of 5A results in a marked and significant increase in the number of cells containing Rad51 foci (Figure 8B) and the percent of cells with >10 Rad51 foci per cell (Supplementary Figure S4B) 2, 4 and 8 h following treatment with 8 Gy of IR compared to V3 cells expressing wild-type DNA-PKcs. In both instances, the number of IR-induced RPA and Rad51 foci observed in 5A cells was similar to the number observed in the uncomplemented DNA-PKcs deficient V3 cell line. Together, the data show blocking autophosphorylation of DNA-PKcs at the serine 2056 phosphorylation cluster results in a decrease in NHEJ and subsequently an increase in IR-induced DNA end resection and recruitment of the HR factor Rad51 to DSBs. Lastly, the data implicate that BRCA1 directly attenuates autophosphorylation of DNA-PKcs at the serine 2056 cluster in S phase of the cell cycle and that this may be a mechanism responsible for DSB repair pathway choice from NHEJ to HR-mediated repair.

## DISCUSSION

Yeast and rodent model system have long suggested that direct competition between HR and NHEJ factors for DSBs is important for the choice between the two pathways (13). Also, increasing evidence has shown that HR may primarily only be active in S phase (41). Thus, in this study we aimed to test if the human canonical NHEJ factors localize to DSBs in S phase and if they could, subsequently identify a mechanism that attenuates NHEJ to allow HR-mediated DSB repair. Our results clearly show that the canonical NHEJ factors are recruited to DSBs in S phase. This is consistent with our previous data showing the kinetics of Ku at DSBs are similar in S and non-S phase cells and a separate report that shows Ku forms IR-induced foci in all cell-cycle phases (30,42). Collectively, the data suggests that DSB pathway choice in humans is more complex than just simple competition between the pathways and that there are likely a number of steps required for pathway choice.

NHEJ is the dominant repair pathway in G1 and G2 even when both NHEJ and HR are functional with the shift from NHEJ to HR is gradual with the highest proportion of breaks repaired by HR in mid-S phase (41). Since the dynamics of Ku70/80 and DNA-PKcs at DSBs is identical in all cell-cycle phases, we speculated there is likely a mechanism which specifically and directly attenuates NHEJ to allow HR to proceed in S phase. DNA-PKcs autophosphorylation at serine 2056 is cell-cycle regulated with phosphorylation at this site high in G1 but markedly attenuated in S phase, implicating that attenuating 2056 phosphorylation may modulate NHEJ specifically in S phase (10). Therefore, we tested if this is the mechanism that modulates NHEJ in S phase and aimed to determine the mechanism mediating this regulation. We found that the pro-HR factor, BRCA1, plays an active role in attenuating the autophosphorylation of DNA-PKcs at the serine 2056 cluster in S phase. We believe that the observed increase in DNA-PKcs autophosphorylation in the BRCA1-defective cell line HCC1937 is not due to an overall defect in HR as we observe an increase in phosphorylation at an early time point (30 min), which is before DNA resection has likely initiated. Furthermore, a previous study found no difference in etoposide or IR-induced threonine 2609 focus formation in HCC1937 cells, suggesting that BRCA1 likely only modulates phosphorylation of DNA-PKcs at serine 2056 and not threonine 2609 (43). The interaction between BRCA1 and DNA-PKcs was found to be specific, peaks in S phase and is mediated by the C-terminal tandem BRCT domain of BRCA1. BRCT domains exist in a number of proteins, including a large number of proteins implicated in the response to DNA damage and are a known protein–protein interaction domain that mediates interactions via its ability to bind the phosphorylated peptide motif pS-P-T-F (36,44,45). The tandem BRCT domain of BRCA1 mediates a number of these types of interactions with a variety of DSB responsive proteins. Surprisingly, DNA-PKcs was found to interact with the tandem BRCT domain of BRCA1 in a phosphorylation-independent manner. A protein interaction screen identified a number of proteins which interact with the tandem BRCT domains of BRCA1 in a phospho-independent manner suggesting that the tandem BRCT domain of BRCA1 can also mediate phospho-independent interactions, such as its interaction with DNA-PKcs (46). The ability of BRCA1 to attenuate autophosphorylation of DNA-PKcs is not mediated by disrupting the ability of DNA-PKcs to localize to or its dynamics at DSBs or via modulation of the overall activity of DNA-PKcs. We found that BRCA1 interacts with the region at or near serine 2056 and directly blocking the ability of DNA-PKcs to autophosphorylate itself.

A possible role for BRCA1 in regulating NHEJ has been tested for a number of years. The data have been conflicting with a number of studies showing BRCA1 plays a negative, positive or no role in NHEJ (47). This is not the first report of BRCA1 interacting with a subunit of the DNA-PK complex. Two studies have shown that BRCA1 interacts with the Ku80 subunit and that they may play a role in regulating each other. One study found that the rapid recruitment of BRCA1 to laser-generated DSBs is dependent on its association with Ku80 (32). Furthermore, it was recently shown that knock-down of BRCA1 suppresses NHEJ repair specifically in the G1 phase of the cell cycle and that BRCA1 promotes high fidelity end-joining of DSBs by stabilizing Ku80 at DSBs during G1 (33). The interaction between BRCA1 and the subunits of the DNA-PK complex are likely distinct as Ku80 interacts with the N-terminal region of the protein, whereas we found that DNA-PKcs interacts with the C-terminal region of BRCA1. Furthermore, we found that DNA-PKcs interacts with BRCA1 in the absence of Ku70/80. Collectively, the data may explain why BRCA1 has been shown to positively and negatively regulate NHEJ as it may influence NHEJ depending on the phase of the cell cycle. However, it should be pointed out that recent data has shown that the pro-HR factors BRCA1/CtIP and the pro-NHEJ Rif1/53BP1 reciprocally regulate each other with BRCA1/CtIP promoting the removal of Rif1/53BP1 from DSBs in S phase and Rif1/53BP1 blocking the ability of BRCA1 to localize to DSBs in G1 (23,24). Therefore, the ability of BRCA1 to positively regulate NHEJ is still under question.

DNA-PKcs is phosphorylated at a number of sites in response to DSBs with the best studied of these being the serine 2056 and threonine 2609 phosphorylation clusters. The serine 2056 cluster is mediated by autophosphorylation, whereas the threonine 2609 cluster is phosphorylated by Ataxia telangiectasia mutated (ATM) and ATM and Rad3 related (ATR) suggesting that the two clusters are distinct (10,39,48). Further evidence that the two clusters are distinct from each other is that they may have opposing roles in regards to regulating the processing of DNA ends (12). Ablation of phosphorylation at the T2609 cluster in a knock-in mouse model results in a marked decrease in NHEJ, hypersensitivity to DNA cross-linking agents and defects in both HR and the Fanconia anemia DNA damage response pathways (49). Furthermore, expression of a phosphorylation-defective mutant of DNA-PKcs at the threonine 2609 cluster dramatically reduces DSB end resection at all DSBs (20). Here we provide further evidence that the two DNA-PKcs phosphorylation clusters are distinct. We found that blocking autophosphorylation of the serine 2056 clusters results in a marked and significant increase in DNA end processing, which is required for HR-mediated repair. We hypothesize that once serine 2056 has been autophosphorylated that the cell has committed itself to NHEJ-mediated DSB repair. Thus, for HR to occur there is a mechanism to block autophosphorylation of DNA-PKcs at serine 2056 in S phase when HR is the preferred pathway. Collectively, we propose the following model. The DNA-PK complex initially localizes to DSBs in all cell-cycle phases because it plays a universal role in protecting DSB ends and subsequently either mediates NHEJ-mediated repair or is displaced to allow other DSB repair pathways to occur. In G1 phase of the cell cycle, the Ku heterodimer localizes to the DSB where it recruits the rest of the NHEJ machinery including DNA-PKcs. DNA-PKcs autophosphorylates at serine 2056 and the cell has fully committed itself to NHEJ-mediated DSB repair. In S phase of the cell cycle, Ku70/80 localizes to the DSB and recruits DNA-PKcs to the DSB. DNA-PKcs is bound with BRCA1 in this cell-cycle phase, which blocks the ability of DNA-PKcs to autophosphorylate and thus mediate NHEJ and allows HR to occur. Therefore, we propose the ability of BRCA1 to block autophosphorylation of DNA-PKcs at the serine 2056 cluster is a mechanism by which cells push pathway choice to HR-mediated repair in S phase of the cell cycle.

## SUPPLEMENTARY DATA

Supplementary Data are available at NAR Online.

SUPPLEMENTARY DATA
