# Supplementary material for: BRCA1 modulates the autophosphorylation status of DNA-PKcs in S phase of the cell cycle
Source: Nucleic Acids Res. 2014 Sep 15;42(18):11487–501. doi: 10.1093/nar/gku824 (PMC4191403; doi:10.1093/nar/gku824)
Supplement: SUPPLEMENTARY DATA [file supp_42_18_11487__index.html]

BRCA1 modulates the autophosphorylation status of DNA-PKcs in S phase of the cell cycle — BRCA1 modulates the autophosphorylation status of DNA-PKcs in S phase of the cell cycle — BRCA1 modulates the autophosphorylation status of DNA-PKcs in S phase of the cell cycle — SUPPLEMENTARY DATA 

# BRCA1 modulates the autophosphorylation status of DNA-PKcs in S phase of the cell cycle

## SUPPLEMENTARY DATA

**Files in this Data Supplement:**

- SUPPLEMENTARY DATA
