# Supplementary material for: BRCA1 modulates the autophosphorylation status of DNA-PKcs in S phase of the cell cycle
Source: Nucleic Acids Res. 2014 Sep 15;42(18):11487–501. doi: 10.1093/nar/gku824 (PMC4191403; doi:10.1093/nar/gku824)
Supplement: SUPPLEMENTARY DATA [file supp_gku824_nar-01526-x-2014-File010.zip › NAR--01526-00001-2014/Davisetal_SupplementaryDataNAR.doc]

**SUPPLEMENTARY FIGURE LEGENDS**

**Figure S1. Exogenously expressed FLAG-tagged BRCA1 interacts with endogenous DNA-PKcs and the interaction is not IR-induced.** FLAG-tagged full-length BRCA1 was transiently expressed in HeLa cells which were non-irradiated or irradiated with 10 Gy and allowed to recover for 1 hour. Subsequently, the FLAG-tagged BRCA1 was immunoprecipitated (IP) using an antiserum specific for the FLAG-tag and analyzed via Western blot (WB) analysis using anti-DNA-PKcs or anti-FLAG antibodies.

**Figure S2.** **BRCA1 does not affect the ability of DNA-PKcs to localize to or its dynamics at DSBs.** Kinetics of relative fluorescent intensity of YFP-tagged DNA-PKcs in U2-OS cells which were treated with non-specific control siRNAs **(A)** or BRCA1 specific siRNAs **(B)** in S and non-S phase cells.

**Figure S3. Ablating phosphorylation of DNA-PKcs at the serine 2056 cluster (5A) results in radiosensitivity. (A)** DNA-PKcs protein expression in V3 cells uncomplemented or complemented with wild-type DNA-PKcs or 5A. Hamster actin levels were analyzed to show equal sample loading. **(B)** Colony formation assays were performed to compare radiation sensitivity of the three V3 cell lines. The cell lines were irradiated at the indicated doses and plated for analysis of survival and colony-forming ability.

**Figure S4. Ablating phosphorylation of DNA-PKcs at the serine 2056 cluster results in an increase in DNA end processing required for homologous recombination.** RPA focus formation following DNA damage is increased in DNA-PKcs deficient (V3) cells complemented with DNA-PKcs in which the serine 2056 cluster is ablated (5A) compared to wild-type complemented cells. Immunostaining of V3 or V3 complemented with wild-type or 5A DNA-PKcs after 8 Gy of γ-ray. Cells were pre-extracted and fixed 2, 4, or 8 hours after irradiation and immunostained for RPA. RPA foci were counted per cell and the percent of cells with >10 RPA foci were quantified. Student t-test was performed to test significance. *= <0.05, **=<0.01. **(B)** Rad51 foci formation following DNA damage is increased in DNA-PKcs deficient (V3) cells complemented with DNA-PKcs in which the serine 2056 cluster is ablated (5A) compared to wild-type complemented cells. Immunostaining of V3 or V3 complemented with wild-type or 5A DNA-PKcs after 8 Gy of γ-ray. Cells were pre-extracted and fixed 2, 4, or 8 hours after irradiation and immunostained for Rad51. Rad51 foci were counted per cell and the percent of cells with >10 Rad51 foci were quantified. Student t-test was performed to test significance. *= <0.05, **=<0.01.
